# Serotonin modifies the impact of sleep disturbance on suicidality in patients with acute coronary syndrome

**DOI:** 10.3389/fpsyt.2022.1046715

**Published:** 2022-11-14

**Authors:** Jae-Min Kim, Ju-Wan Kim, Hee-Ju Kang, Wonsuk Choi, Seunghyong Ryu, Ju-Yeon Lee, Sung-Wan Kim, Jung-Chul Kim, Byung Jo Chun, Il-Seon Shin, Youngkeun Ahn, Myung Ho Jeong

**Affiliations:** ^1^Department of Psychiatry, Chonnam National University Medical School, Gwangju, South Korea; ^2^Department of Internal Medicine, Chonnam National University Hwasun Hospital, Chonnam National University Medical School, Hwasun, South Korea; ^3^Department of Surgery, Chonnam National University Medical School and Hospital, Gwangju, South Korea; ^4^Department of Emergency Medicine, Chonnam National University Medical School, Gwangju, South Korea; ^5^Department of Cardiology, Chonnam National University Medical School, Gwangju, South Korea

**Keywords:** serotonin, sleep, modifying effect, suicidality, acute coronary syndrome

## Abstract

**Background:**

This study investigated the associations of sleep disturbance and serum serotonin levels with suicidal ideation, and evaluated the potential modifying effects of serotonin on these associations in patients with the acute coronary syndrome (ACS).

**Methods:**

In total, 969 ACS patients were recruited from a tertiary university hospital in Korea within 2 weeks of disease onset and evaluated in terms of sleep disturbance (using the Leeds Sleep Evaluation Questionnaire), serum serotonin levels, and suicidal ideation (using the “suicidal thoughts” item of the Montgomery–Åsberg Depression Rating Scale). Covariates included sociodemographics, depression, vascular risk factors, and disease severity. After 1 year, 711 patients were re-evaluated in terms of suicidal ideation. Logistic regression analysis was performed with adjustment for covariates.

**Results:**

Sleep disturbance was significantly associated with suicidal ideation at baseline and follow-up. Serum serotonin showed no such association but modified the association of sleep disturbance with suicidal ideation such that it was significant only in the lower serum serotonin group, with significant interaction terms obtained after adjustment for relevant covariates.

**Conclusion:**

Evaluating sleep disturbance and serum serotonin levels could improve the accuracy of clinical predictions of suicidal ideation in the acute and chronic phases of ACS.

## Introduction

Acute coronary syndrome (ACS) is a leading cause of morbidity and mortality worldwide (1). Patients experiencing this kind of life-threatening disease are at an increased risk for suicidality (2). Suicidal ideation is a precondition of more serious suicidal behavior including suicidal attempt or completion but could be a treatment target with appropriate detection (3). Identifying potential determinants of suicidality might be the initial step.

The etiology of suicidal behavior is complex, and various biopsychosocial factors have been investigated in association with suicide risk (4). Risk factors for suicidal behavior are suggested to be at least partially heritable (about 40–50%), including genetic variants that have been implicated in the pathogenesis of this complex phenomenon (5). As a possible contributor to negative emotions and negative clinical outcomes, patients with affective temperamental dysregulation are more likely to have higher feelings of hopelessness, an important predictor of suicidal potential (6). One growing body of research is the associations with sleep disturbances. Sleep disturbance is increasingly being studied in association with suicidal behavior. Both sleep disorders and general sleep complaints were found to be related to higher levels of suicidal ideation and behaviors in a meta-analysis (7). A close link between sleep disturbance and suicidality may indicate a common biological basis (8). Serotonin appears to be a candidate because it plays an important role in both sleep regulation and suicidality. Although the relationship between serotonin and sleep is complex, recent studies have suggested that serotonin release during waking states induces homeostatic regulation of slow-wave sleep (9) and that serotonergic dysfunction promotes night-time wakefulness (10). The serotonergic system is involved in both stress and diathesis models of suicidal behaviors (11), and low blood serotonin levels are related to increased suicidality (12). Higher rates of suicidality have been hypothesized to be correlated with reduced sleep duration, possibly mediated by decreased serotonin activity (13). However, the roles of sleep disturbance and serotonin have not been formally tested in patients with ACS, who are at high risk of suicidal behavior.

Considering these lines of evidence of associations among sleep problems, affective dysregulation, and suicidality, and close biological relationships between serotonin and sleep problems and suicidality, we hypothesized that serotonin might modify the harmful effects of sleep disturbance in terms of suicidal behavior in patients with ACS. Using data from a prospective Korean cohort with ACS, we investigated associations among sleep disturbance, serum serotonin levels measured in the acute phase of ACS, and suicidal ideation evaluated in both the acute and chronic phases, and then evaluated the potential modifying effects of serum serotonin levels on the association between sleep disturbance and suicidal ideation.

## Materials and methods

### Study outline and participants

The objectives of this observational sub-study (part of the K-DEPACS study) (14) were to investigate the occurrence, risk factors, and longitudinal course of mental disorders in survivors of recently developed ACS, and the impacts of psychiatric problems on the cardiac course and prognosis. In brief, patients with recent ACS were approached at the Department of Cardiology of Chonnam National University Hospital, Gwangju, South Korea, from 2006 to 2012 and invited to participate in this study. Participants who met the eligibility criteria (see Supplementary material) and agreed to participate (*N* = 1,152) were examined as inpatients at the baseline evaluation (e.g., questionnaires on sleep and suicidal ideation) after critical care unit treatment, within 2 weeks (mean ± standard deviation = 6.3 ± 2.4 days) of ACS onset. Thereafter, assessments were performed within 2 weeks (baseline) in hospitalized patients, and at 1 year (follow-up) in outpatients, to investigate disease consequences in the acute and chronic phases. Sleep disturbance and serum serotonin levels were estimated at baseline, and suicidal ideation was evaluated in both phases. This study was approved by the Chonnam National University Hospital Institutional Review Board. All participants reviewed the consent form and provided written informed consent.

### Sleep disturbances

Sleep disturbances were assessed using the Leeds Sleep Evaluation Questionnaire (LSEQ) (15). The LSEQ is composed of 10 self-reported items each of which is scored on a 100-mm visual analog scale. Visual analog scales consist of a 100-ram-horizontal line with two extreme states defined at the ends of the line (e.g., more difficult than usual = score of 0, Easier than usual = score of 10). These items are related to the ease of getting to sleep (GTS), quality of sleep (QOS), ease of awakening from sleep (AFS), and alertness and behavior following wakefulness (BFW). The time frame of the LSEQ for evaluation of sleep problems was the most recent 1 week after ACS, as recommended by the scale developer.

### Serum serotonin

Participants were instructed to fast overnight before blood sampling and were asked to sit quietly for 25–45 min before blood samples were drawn. Serum serotonin levels were measured using the ClinRep high-performance liquid chromatography kit (Recipe, Munich, Germany) at GreenCross LabCell (Yongin, Korea).

### Suicidal ideation

Suicidal ideation was identified using the “suicidal thoughts” item of the Montgomery–Åsberg Depression Rating Scale (MADRS-ST) (16), which consists of 10 items rated for severity on an ordinal scale ranging from 0 to 6. The MADRS is used extensively worldwide in clinical research to assess the severity of depression and has good reliability and validity. The “suicidal thoughts” item assesses the feeling that life is not worth living and plans for suicide, with scores ranging between 0 (*life satisfaction*) and 6 (*explicit plans for suicide*). Suicidal ideation was considered to be present if the patient scored ≥ 2, where a score of 2 reflected fleeting suicidal thoughts, following previous studies (17, 18).

### Baseline covariates

Sociodemographic characteristics of interest included age, sex, duration of education, living status (living alone or not), housing status (owned or rented), and current occupation (employed or not). Depression-related characteristics included previous and family history of depression, and current DSM-IV depressive disorder (19). The following vascular risk factors were evaluated: previous and family history of ACS or stroke, diagnosis of hypertension or diabetes mellitus, hypercholesterolemia (defined as fasting serum total cholesterol > 200 mg/dL), obesity (defined as body mass index > 25 kg/m^2^), and current smoking status. To evaluate current cardiac status, ACS severity was estimated using the Killip classification (20), the left ventricular ejection fraction was estimated based on echocardiography results, and the serum cardiac biomarkers troponin I and creatine kinase-MB were measured.

### Statistical analyses

Potential baseline covariates were analyzed according to sleep status and suicidal ideation at baseline and follow-up using Student’s *t*-tests or χ^2^ tests, as appropriate. Covariates for further adjusted analyses were selected from among the characteristics significantly associated with sleep disturbance and suicidal ideation (*P*< 0.05), and other variables with potential effects on suicidal behaviors (3, 18), with consideration of the potential for collinearity between the variables. Correlations between sleep disturbance and serum serotonin were estimated (Spearman’s ρ). The individual associations of sleep disturbance (lower vs. higher) and serum serotonin (higher vs. lower) with suicidal ideation were analyzed at both baseline and follow-up using logistic regression models, before and after adjusting for covariates. Modifying effects of serum serotonin levels on these associations were analyzed using multinomial logistic regression with adjustment for the same factors. All statistical tests were two-sided, with a significance level of *P* < 0.05. Statistical analyses were performed using SPSS 27.0 software (IBM Corp., Armonk, NY, USA).

## Results

### Recruitment and descriptive data

Participant recruitment and prevalence rates of suicidal ideation are shown in Supplementary Figure 1. A total of 969 ACS patients who met the eligibility criteria and agreed to offer blood samples comprised the baseline sample. Of these participants, 711 (73%) who were successfully re-evaluated 1 year later comprised the follow-up sample.

Baseline characteristics were compared according to the LSEQ GTS scores (lower vs. higher) (Table 1). Age, sex, education, rates of living alone, current employment, previous and family history of depression, present clinical depression, hypertension, diabetes, and smoking were significantly different between the groups. Suicidal ideation was present in 195 (20%) and 87 (12%) patients with ACS at baseline and follow-up, respectively. The baseline characteristics of patients with and without suicidal ideation at baseline and follow-up are listed in Supplementary Table 1. Suicidal ideation at baseline was significantly associated with female sex, lower education level, rented housing, current unemployment, and previous and present depression, whereas suicidal ideation at follow-up was significantly associated with female sex, family history, and present depression. Considering these and previous findings (3, 21), and the collinearity between variables, 10 covariates were selected for subsequent adjusted analysis: sex, education, housing, current employment, previous and family history of depression, present clinical depression, hypertension, diabetes, and serum troponin I levels.

**TABLE 1:** Baseline characteristics by ease of getting to sleep (GTS) as assessed in the Leeds Sleep Evaluation Questionnaire (LSEQ) at 2 weeks after acute coronary syndrome (ACS).

	Lower GTS (*N* = 586)	Higher GTS (*N* = 383)	Statistical coefficient	*P*-value
**Socio-demographic characteristics**				
Age, mean (SD) years	56.9 (11.0)	60.2 (10.9)	*t* = -4.669	**<0.001**
Sex, *N* (%) female	127 (21.7)	142 (37.1)	χ^2^ = 27.403	**<0.001**
Education, mean (SD) years	10.4 (4.6)	9.0 (4.6)	*t* = + 4.437	**<0.001**
Living alone, *N* (%)	41 (7.0)	51 (13.3)	χ^2^ = 10.764	**0.001**
Housing, *N* (%) rented	85 (14.5)	65 (17.0)	χ^2^ = 1.077	0.299
Currently unemployed, *N* (%)	185 (31.6)	183 (47.8)	χ^2^ = 25.841	**<0.001**
**Depression characteristics, *N* (%)**				
Previous depression	13 (2.2)	21 (5.5)	χ^2^ = 7.291	**0.007**
Family history of depression	8 (1.4)	15 (3.9)	χ^2^ = 6.506	**0.011**
Present clinical depression	41 (7.0)	136 (35.5)	χ^2^ = 178.454	**<0.001**
**Vascular risk factors, *N* (%)**				
Previous ACS	29 (4.9)	10 (2.6)	χ^2^ = 3.277	0.070
Family history of ACS	19 (3.2)	12 (3.1)	χ^2^ = 0.009	0.925
Hypertension	260 (44.4)	198 (51.7)	χ^2^ = 4.991	**0.025**
Diabetes mellitus	97 (16.6)	94 (24.5)	χ^2^ = 9.344	**0.002**
Hypercholesterolemia	286 (48.8)	200 (52.2)	χ^2^ = 1.080	0.299
Obesity	256 (43.7)	159 (41.5)	χ^2^ = 0.446	0.504
Current smoker	245 (41.8)	121 (31.6)	χ^2^ = 10.285	**0.001**
**Current cardiac status**				
Killip class > 1, *N* (%)	96 (16.4)	72 (18.8)	χ^2^ = 0.944	0.331
Left ventricular ejection fraction, mean (SD)%	61.4 (11.1)	60.9 (11.5)	*t* = + 0.681	0.496
Troponin I, mean (SD) mg/dL	9.9 (15.7)	9.8 (13.6)	*t* = + 0.119	0.905
Creatine kinase -MB, mean (SD) mg/dL	16.2 (33.9)	19.2 (42.0)	*t* = -1.194	0.233

Bold style indicates statistical significance (*p*-value < 0.05).

### Individual associations with suicidal ideation

Serum serotonin levels were negatively correlated with GTS (Spearman’s ρ = −0.0.063; *P* = 0.048) and AFS (Spearman’s ρ = −0.064; *P* = 0.045), but not with QOS or BFS (*P*> 0.1 for both). The individual associations of the four factors of LSEQ with suicidal ideation are described in Table 2. All four factors were significantly associated with suicidal ideation at baseline before and after adjustment. At follow-up, QOS and AFS were significantly associated with suicidal ideation before and after adjustment, whereas GTS and BFS showed no significant association after adjustment. No association was found between serum serotonin and suicidal ideation before or after adjustment (*P*> 0.3 for all).

**TABLE 2:** Associations of four factors of the Leeds Sleep Evaluation Questionnaire (LSEQ) with suicidal ideation (SI) in patients with acute coronary syndrome (ACS).

LSEQ	Group	Suicidal ideation at baseline (*N* = 969)				Suicidal ideation at follow-up (*N* = 711)			
			
		*N*	No. (%) presence	OR (95% CI)		*N*	No. (%) presence	OR (95% CI)	
							
				Unadjusted	Adjusted			Unadjusted	Adjusted
Ease of getting to sleep	Lower	586	84 (14.3)	1.00	1.00	423	41 (12.5)	1.00	1.00
	Higher	383	111 (29.0)	2.44 (1.77–3.36)^‡^	2.14 (1.54–2.98)^‡^	288	46 (16.0)	1.77 (1.13–2.78)*	1.41 (0.87–2.29)
Quality of sleep	Lower	554	69 (12.5)	1.00	1.00	401	37 (9.2)	1.00	1.00
	Higher	415	126 (30.4)	3.07 (2.21–4.25)^‡^	2.89 (2.06–4.04)^‡^	310	50 (16.1)	1.89 (1.20–2.98)^†^	1.61 (1.00–2.59)*
Ease of awakening in the morning from sleep	Lower	658	103 (15.7)	1.00	1.00	482	47 (9.8)	1.00	1.00
	Higher	311	92 (29.6)	2.26 (1.64–3.12)^‡^	2.17 (1.55–3.04)^‡^	229	40 (17.5)	1.96 (1.24–3.09)^†^	1.72 (1.07–2.77)*
Integrity of behavior following wakefulness	Lower	482	71 (14.7)	1.00	1.00	353	33 (9.3)	1.00	1.00
	Higher	487	124 (25.5)	1.98 (1.43–2.73)^‡^	1.87 (1.34–2.61)^‡^	358	54 (15.1)	1.72 (1.09–2.73)*	1.54 (0.96–2.47)

Adjusted ORs (95% CIs) were estimated adjustment for sex, education, housing, current employment, previous and family history of depression, present clinical depression, hypertension, diabetes, and serum troponin I levels. *P < 0.05; **^†^***P* < 0.01; ^‡^*P* < 0.001.

### Modifying effects of serum serotonin

**FIGURE 1:**
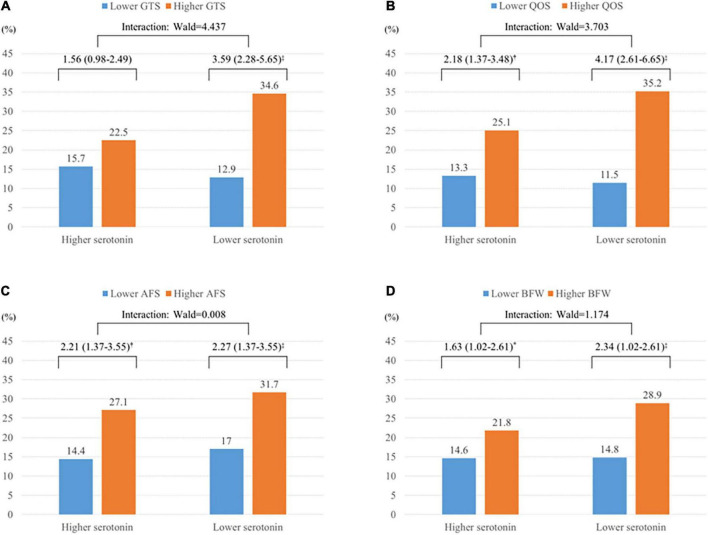
Modifying effects of serum serotonin on the associations between four factors of the Leeds Sleep Evaluation Questionnaire (LSEQ) and suicidal ideation at baseline in patients with acute coronary syndrome (ACS) (*N* = 969). **(A)** Ease of getting to sleep (GTS). **(B)** Quality of sleep (QOS). **(C)** Ease of awakening in the morning from sleep (AFS). **(D)** Integrity of behavior following wakefulness (BFW). Data are odds ratios (95% confidence intervals) adjusted for sex, education, housing, current employment, previous and family history of depression, present clinical depression, hypertension, diabetes, and serum troponin I levels. *P < 0.05; **^†^***P* < 0.01; ^‡^*P* < 0.001.

**FIGURE 2:**
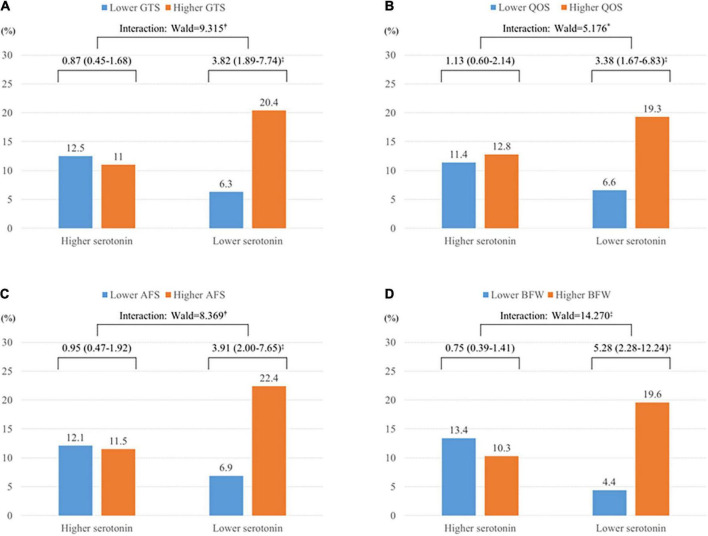
Modifying effects of serum serotonin on the associations between four LSEQ factors and suicidal ideation at follow-up in patients with ACS (*N* = 711). **(A)** Ease of getting to sleep (GTS). **(B)** Quality of sleep (QOS). **(C)** Ease of awakening in the morning from sleep (AFS). **(D)** Integrity of behavior following wakefulness (BFW). Data are odds ratios (95% confidence intervals) adjusted for sex, education, housing, current employment, previous and family history of depression, present clinical depression, hypertension, diabetes, and serum troponin I levels. *P < 0.05; **^†^***P* < 0.01; ^‡^*P* < 0.001.

The modulatory effects of serum serotonin on the association between sleep disturbance and suicidal ideation at baseline and follow-up are shown in Figures 1, 2, respectively. Worse sleep status was significantly associated with suicidal ideation at baseline in both the higher and lower serotonin groups; there were no significant interaction terms in the adjusted analysis (Figure 1). However, the association between sleep disturbance and suicidal ideation at follow-up was significant only in the lower serum serotonin group, with significant interaction terms seen after adjustment (Figure 2).

## Discussion

In this longitudinal study of ACS patients, sleep disturbances were significantly associated with suicidal ideation at baseline and follow-up. Although serum serotonin levels were not associated with suicidal ideation, it modified the long-term effects, in that the associations of sleep disturbances and suicidal ideation at follow-up were significant only in the lower but not in the higher serum serotonin group with significant interaction terms after adjustment for relevant covariates.

Various sleep problems including insomnia, sleep-disordered breathing, nightmare, and subjective sleep complaints have been consistently associated with suicidal behaviors in various populations (7). Our findings confirmed the well-known association even in these patients with ACS both at acute and chronic phases. In particular, subjective sleep complaints rather than sleep disorders were used for exposure variables, but all four factors of subjective disturbances showed significant associations with suicidal ideation. The strengths of the observations remained significant after adjustment for relevant covariates including depressive variables, also keeping with previous study results on the significant association beyond depressive status. Our results underscore the importance of evaluating sleep disturbances in the early stage of ACS considering its associations with suicidal ideation both at acute and chronic phases.

Various lines of evidence point to the central role of serotonin in suicide. Abnormalities in number of serotonergic neurons, serotonin transportation, receptor binding and serotonin levels in key brain areas have all been linked with suicide (11). Functional imaging studies have demonstrated decreased serotonin transporter binding in attempted suicide (22) while studies using quantitative receptor autoradiography have noted higher 5HT1A receptor binding in brain stem and prefrontal cortex (23) Mann Serotonin deficiency was linked to decision-making and impulsive aggressive traits related to suicidal intent (11), and low blood serotonin levels were associated with suicidal behavior (12). However, no such associations were found in this study. The discrepancy in the study population (general population vs. ACS patients) and suicidality definition (suicidal attempt vs. suicidal ideation) might account for the different observations. Also, previous studies have shown that genetic mutations related to serotonin are associated with suicide. In our report, gene variants were not assessed for further examination of the suicidal ideas. It appears that genetic and epigenetic factors may be key to understanding the molecular mechanisms that underlie an individual’s vulnerability to suicide.

Despite no direct impact, significant modifying effects of serum serotonin levels were found on the associations between sleep disturbances and suicidal ideation. Several pathomechanisms are plausible. First, stress-diathesis models are the well-accepted theory in the pathophysiology of suicidal behaviors (4). The negative impacts of sleep disturbances (stress) could be aggravated in the presence of lower serotonin levels (diathesis) on suicidal ideation in these participants. Second, the possible relationship between serotonin and disturbances might be considered. That is, serotonin levels were significantly and negatively correlated to the GTS and AFS factors of the LSEQ in this study, in line with previous literature on the connections between the serotonergic system and sleep disturbances (7, 10). Hence, the two unfavorable statuses arising from mutual influences might have synergistic effects on suicidality. Third, serotonin has been considered a common biomarker for both sleep regulation and suicidality (8).

It is noteworthy that the modifying effects of serotonin were evident only on suicidal ideation at 1-year follow-up but not on that at baseline. In this study, baseline evaluations were made within 2 weeks after ACS events at the hospital. In this acute phase, treatment is focused on intensive medical care with certain procedures and patients are usually under overwhelming and unstable emotional status. All these conditions are unfavorable for sleep duration and quality. Therefore, modifying effects of serotonin could be weakened at this acute phase.

The limitations of this study were as follows. The main dependent and outcome variable was suicidal ideation; although suicidal ideation is closely related to more severe suicidal behavior (3), no data concerning suicidal attempts or completion were obtained. However, previous clinical studies of patients with ACS or other severe physical disorders also used suicidal ideation as a phenotype, because more severe forms of suicidal behavior are rare in this type of study (17, 21). Suicidal ideation was identified using the “suicidal thoughts” item of the MADRS rather than a specific instrument. However, this method was used in previous studies of patients with severe physical disorders (15). Moreover, a recent study claimed that a depression rating scale item about suicidal thoughts had good validity (24). Recruitment was carried out at a single hospital, which may limit the generalizability of our findings; however, this may also be a strength as it increased the consistency and reliability of the evaluations. Finally, correlation analysis of peripheral blood and cerebrospinal fluid serotonin levels is inconclusive (25), although the results of this and a previous study (12) indicated that peripheral blood serotonin levels could be used as a biomarker for suicidality.

## Conclusion

In conclusion, we found that lower serotonin levels at baseline strengthened the well-known association between sleep problems at baseline and suicidal ideation 1 year after ACS onset. With respect to pathophysiology, our findings provide insight into the pathogenic effects of sleep disturbance on suicidality, suggesting a modifying role of serotonin. In clinical practice, the prediction of suicidality could be improved by evaluating sleep disturbance and serum serotonin levels in the acute phase of ACS. Because ACS is a leading cause of morbidity and mortality, this procedure could significantly reduce the disease burden, which is particularly important in cardiovascular clinics (because interviews to evaluate suicidality can be difficult for untrained clinicians). In addition, exercise is associated with a reduction in cardiac mortality, and is effective as a non-pharmacological treatment for depression and sleep disorders; therefore, exercise can serve as an initial actionable recommendation for ACS patients, augmenting first-line treatments with minimal adverse effects (26, 27). This study represents an important first step toward fully elucidating the modulatory role of serotonin in the association between sleep disturbances and suicidality. Future multi-center studies in other settings are needed to confirm the generalizability of the present findings.

The English in this document has been checked by at least two professional editors, both native speakers of English. For a certificate, please see: http://www.textcheck.com/certificate/zMYaSJ.

## Data availability statement

The original contributions presented in this study are included in the article/Supplementary material, further inquiries can be directed to the corresponding author.

## Ethics statement

The studies were approved separately by the Chonnam National University Hospital Institutional Review Board (CNUH-2012-014). The patients/participants provided their written informed consent to participate in this study.

## Author contributions

J-MK designed the study. J-WK, S-WK, and I-SS construct and performed study methodology. H-JK, J-CK, SR, and J-YL contributed to project administration. YA and MJ contributed to validation. J-MK, H-JK, J-WK, S-WK, and BC acquired and curated the data. J-MK, J-WK, H-JK, and I-SS contributed to formal analysis. J-MK and J-WK contributed to writing—original draft. J-MK, H-JK, J-WK, SR, and WC contributed to reviewing and editing of draft. All authors contributed to the article and approved the submitted version.

## References

[1] Virani SS Alonso A Benjamin EJ Bittencourt MS Callaway CW Carson AP Heart disease and stroke statistics 2020 update: a report from the American Heart Association. *Circulation.* (2020) 141:e139–596. 31992061 10.1161/CIR.0000000000000757

[2] Larsen KK Agerbo E Christensen B Sondergaard J Vestergaard M. Myocardial infarction and risk of suicide: a population-based case-control study. *Circulation.* (2010) 122:2388–93. 21098443 10.1161/CIRCULATIONAHA.110.956136

[3] Kim JM Kang HJ Bae KY Kim SW Shin IS Hong YJ Determinants and escitalopram treatment effects on suicidal ideation in patients with acute coronary syndrome: findings from the K-DEPACS and EsDEPACS studies. *Int J Cardiol.* (2016) 219:225–30. 10.1016/j.ijcard.2016.06.048 27336191

[4] Oquendo MA Sullivan GM Sudol K Baca-Garcia E Stanley BH Sublette ME Toward a biosignature for suicide. *Am J Psychiatry.* (2014) 171:1259–77. 25263730 10.1176/appi.ajp.2014.14020194 PMC4356635

[5] Mcguffin P Marusic A Farmer A. What can psychiatric genetics offer suicidology? *Crisis.* (2001) 22:61–5. 11727895 10.1027//0227-5910.22.2.61

[6] Serafini G Pompili M Innamorati M Gentile G Borro M Lamis DA Gene variants with suicidal risk in a sample of subjects with chronic migraine and affective temperamental dysregulation. *Eur Rev Med Pharmacol Sci.* (2012) 16:1389–98. 23104655

[7] Harris LM Huang X Linthicum KP Bryen CP Ribeiro JD. Sleep disturbances as risk factors for suicidal thoughts and behaviours: a meta-analysis of longitudinal studies. *Sci Rep.* (2020) 10:13888. 10.1038/s41598-020-70866-6 32807889 PMC7431543

[8] Bernert RA Joiner TE. Sleep disturbances and suicide risk: a review of the literature. *Neuropsychiatr Dis Treat.* (2007) 3:735–43. 10.2147/ndt.s1248 19300608 PMC2656315

[9] Jouvet M. Sleep and serotonin: an unfinished story. *Neuropsychopharmacology.* (1999) 21:24–7s. 10.1016/S0893-133X(99)00009-3 10432485

[10] Ursin R. Serotonin and sleep. *Sleep Med Rev.* (2002) 6:57–69. 10.1053/smrv.2001.0174 12531142

[11] Mann JJ. The serotonergic system in mood disorders and suicidal behaviour. *Philos Trans R Soc Lond B Biol Sci.* (2013) 368:20120537. 10.1098/rstb.2012.0537 23440471 PMC3638390

[12] Tyano S Zalsman G Ofek H Blum I Apter A Wolovik L Plasma serotonin levels and suicidal behavior in adolescents. *Eur Neuropsychopharmacol.* (2006) 16:49–57. 10.1016/j.euroneuro.2005.05.005 16076550

[13] Kim JM Bae KY Kang HJ Kim SW Shin IS Hong YJ Design and methodology for the Korean observational and escitalopram treatment studies of depression in acute coronary syndrome: K-DEPACS and EsDEPACS. *Psychiatry Investig.* (2014) 11:89–94. 10.4306/pi.2014.11.1.89 24605129 PMC3942557

[14] Parrot AC Hindmarch I. The leeds sleep evaluation questionnaire in psychopharmacological investigation-a review. *Psychopharmacology.* (1980) 71:173–9. 10.1007/BF00434408 6777817

[15] Montgomery SA Asberg M. A new depression scale designed to be sensitive to change. *Br J Psychiatry.* (1979) 134:382–9. 444788 10.1192/bjp.134.4.382

[16] Santos CO Caeiro L Ferro JM Figueira ML. A study of suicidal thoughts in acute stroke patients. *J Stroke Cerebrovasc Dis.* (2012) 21:749–54. 22000522 10.1016/j.jstrokecerebrovasdis.2011.04.001

[17] Murrough JW Soleimani L DeWilde KE Collins KA Lapidus KA Iacoviello BM Ketamine for rapid reduction of suicidal ideation: a randomized controlled trial. *Psychol Med.* (2015) 45:3580. 10.1017/S0033291715001506 26266877

[18] American Psychiatric Association. *Diagnostic and Statistical Manual of Mental Disorders.* 4th ed. Virginia, VA: American Psychiatric Association (1994).

[19] Killip T Kimball JT. Treatment of myocardial infarction in a coronary care unit: a two year experience with 250 patients. *Am J Cardiol.* (1967) 20:457–64. 10.1016/0002-9149(67)90023-9 6059183

[20] Nascimento ER Maia AC Soares-Filho G Nardi AE Cardoso A. Predictors of suicidal ideation in coronary artery disease. *Compr Psychiatry.* (2015) 57:16–20. 25464838 10.1016/j.comppsych.2014.10.017

[21] Miller JM Hesselgrave N Ogden RT Sullivan GM Oquendo MA Mann JJ Positron emission tomography quantification of serotonin transporter in suicide attempters with major depressive disorder. *Biol Psychiatry.* (2013) 74:287–95. 10.1016/j.biopsych.2013.01.024 23453288 PMC3725207

[22] Arango V Underwood MD Mann JJ. Serotonin brain circuits involved in major depression and suicide. *Prog Brain Res.* (2002) 136:443–53. 10.1016/s0079-6123(02)36037-0 12143401

[23] Green KL Brown GK Jager-Hyman S Cha J Steer RA Beck AT. The predictive validity of the beck depression inventory suicide item. *J Clin Psychiatry.* (2015) 76:1683–6. 10.4088/JCP.14m09391 26717528

[24] Audhya T Adams JB Johansen L. Correlation of serotonin levels in CSF, platelets, plasma, and urine. *Biochim Biophys Acta.* (2012) 1820:1496–501. 22664303 10.1016/j.bbagen.2012.05.012

[25] Ji H Fang L Yuan L Zhang Q. Effects of exercise-based cardiac rehabilitation in patients with acute coronary syndrome: a meta-analysis. *Med Sci Monit.* (2019) 25:5015–27. 31280281 10.12659/MSM.917362 PMC6636406

[26] Murri MB Ekkekakis P Magagnoli M Zampogna D Cattedra S Capobianco L Physical exercise in major depression: reducing the mortality gap while improving clinical outcomes. *Front Psychiatry.* (2019) 9:762. 10.3389/fpsyt.2018.00762 30687141 PMC6335323

[27] Kohyama J. Sleep, serotonin, and suicide in Japan. *J Physiol Anthropol.* (2011) 30:1–8. 10.2114/jpa2.30.1 21307614

