# Supplementary material for: Serotonin modifies the impact of sleep disturbance on suicidality in patients with acute coronary syndrome
Source: Front Psychiatry. 2022 Nov 14;13:1046715. doi: 10.3389/fpsyt.2022.1046715 (PMC9702559; doi:10.3389/fpsyt.2022.1046715)
Supplement: Supplementary file 1 [file Data_Sheet_1.DOCX]

**Online Supplement**

**Eligibility criteria for patients with acute coronary syndrome (ACS)**

Inclusion criteria were: i) aged 18~85 years; ii) confirmed ACS by investigation (the presence of ST-segment elevation MI was determined by >30 min of continuous chest pain, a new ST-segment elevation ≥2 mm on at least two contiguous electrocardiographic leads, and creatine kinase-MB more than three times normal; the presence of non-ST-segment elevation MI was diagnosed by chest pain and a positive cardiac biochemical marker without new ST-segment elevation; and the presence of unstable angina was determined by chest pain within the preceding 72 h with or without ST-T wave changes or positive cardiac biochemical markers); iii) ability to complete study questionnaires; iv) ability to understand the study objectives and sign informed consent. Exclusion criteria were: i) occurrence of ACS while hospitalized for another reason; ii) ACS developing less than 3 months after a coronary artery bypass graft procedure; iii) uncontrolled hypertension (systolic blood pressure (BP) >180mmHg or diastolic BP >100mmHg); iv) resting heart rate <40/min; v) severe physical illnesses threatening life or interfering with the recovery from ACS; vi) persistent clinically significant laboratory abnormalities in complete blood cell counts, thyroid or renal function tests.

**Fig. S1.**

Recruitment process and prevalence of suicidal ideation (SI) in participants with acute coronary syndrome (ACS).


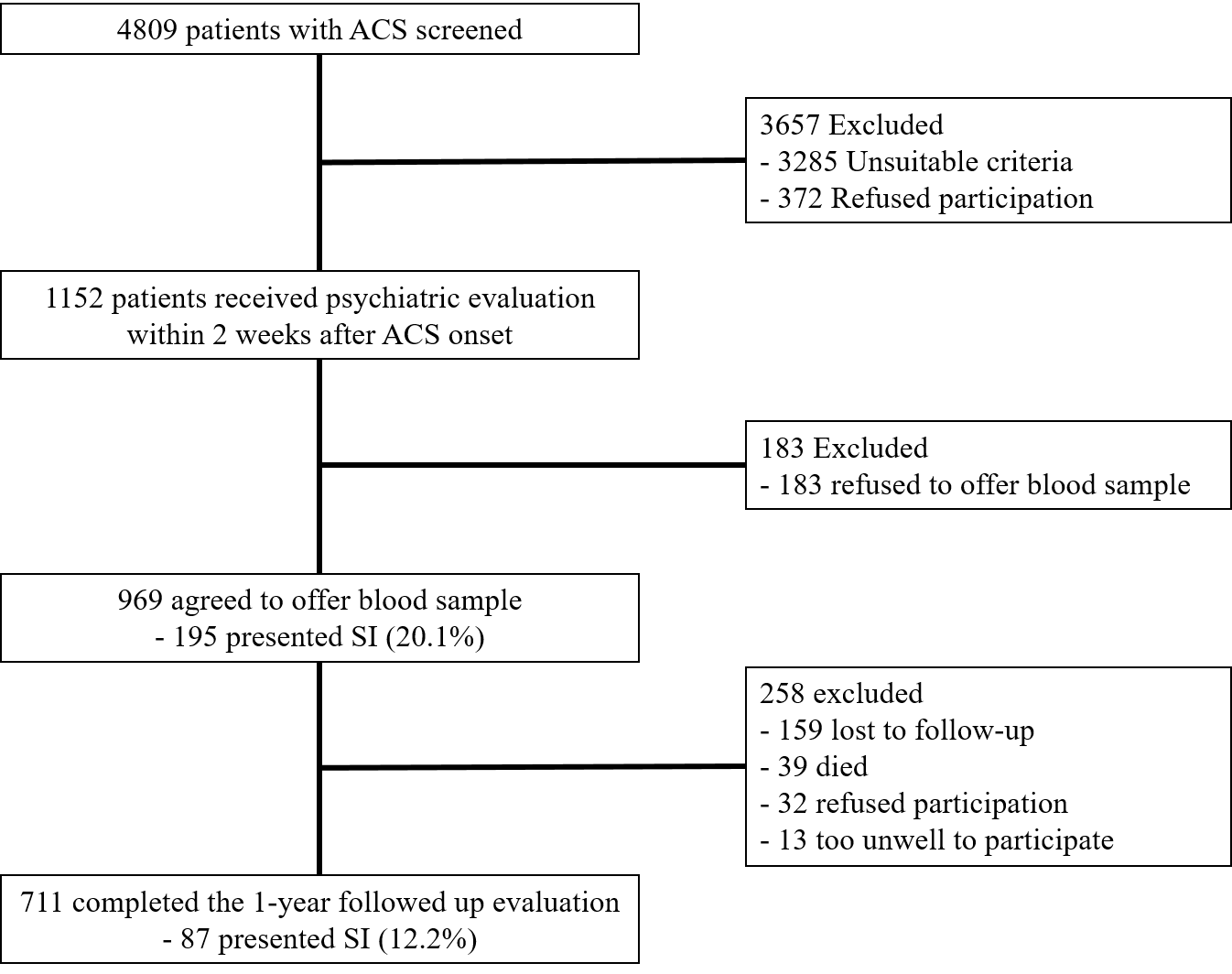


| Table S1. Baseline characteristics by suicidal ideation (SI) status in patients with acute coronary syndrome (ACS). | | | | | | | |
| --- | --- | --- | --- | --- | --- | --- | --- |
|  | Baseline sample (N=969) | | |  | Follow-up sample (N=711) | | |
|  | Absent SI  (N=774) | Present SI  (N=195) | p-value |  | Absent SI  (N=624) | Present SI  (N=87) | p-value |
| **Socio-demographic characteristics** |  |  |  |  |  |  |  |
| Age, mean (SD) years | 58.0 (11.3) | 58.9 (10.6) | 0.315 |  | 57.6 (10.7) | 57.4 (11.1) | 0.855 |
| Sex, N (%) female | 201 (26.0) | 68 (34.9) | **0.013** |  | 163 (26.1) | 33 (37.9) | **0.021** |
| Education, mean (SD) year | 10.0 (4.7) | 9.1 (4.4) | **0.012** |  | 10.0 (4.6) | 9.2 (4.8) | 0.118 |
| Living alone, N (%) yes | 71 (9.2) | 21 (10.8) | 0.497 |  | 49 (7.9) | 11 (12.6) | 0.132 |
| Housing, N (%) rented | 109 (14.1) | 41 (21.0) | **0.017** |  | 102 (16.3) | 20 (23.0) | 0.124 |
| Currently unemployed, N (%) | 279 (36.0) | 89 (45.6) | **0.014** |  | 213 (34.1) | 38 (43.7) | 0.081 |
| **Depression characteristics, N (%)** |  |  |  |  |  |  |  |
| Previous depression | 19 (2.5) | 15 (7.7) | **<0.001** |  | 24 (3.8) | 6 (6.9) | 0.247 |
| Family history of depression | 15 (1.9) | 8 (4.1) | 0.108 |  | 14 (2.2) | 6 (6.9) | **0.026** |
| Present clinical depression | 221 (28.6) | 157 (80.5) | **<0.001** |  | 232 (37.2) | 53 (60.9) | **<0.001** |
| **Vascular risk factors, N (%)** |  |  |  |  |  |  |  |
| Previous ACS | 30 (3.9) | 9 (4.6) | 0.639 |  | 25 (4.0) | 6 (6.9) | 0.255 |
| Family history of ACS | 24 (3.1) | 7 (3.6) | 0.729 |  | 18 (2.9) | 6 (6.9) | 0.103 |
| Hypertension | 360 (46.5) | 98 (50.3) | 0.349 |  | 282 (45.2) | 42 (48.3) | 0.588 |
| Diabetes mellitus | 144 (18.6) | 47 (24.1) | 0.085 |  | 118 (18.9) | 24 (27.6) | 0.058 |
| Hypercholesterolemia | 384 (49.6) | 102 (52.3) | 0.501 |  | 333 (53.4) | 50 (57.5) | 0.472 |
| Obesity | 341 (44.1) | 74 (37.9) | 0.123 |  | 280 (44.9) | 35 (40.2) | 0.414 |
| Current smoker | 297 (38.4) | 69 (35.4) | 0.442 |  | 247 (39.6) | 33 (37.9) | 0.768 |
| **Current cardiac status** |  |  |  |  |  |  |  |
| Killip class >1, N (%) | 132 (17.1) | 36 (18.5) | 0.643 |  | 101 (16.2) | 13 (14.9) | 0.767 |
| Left ventricular ejection fraction, mean (SD) % | 61.2 (11.4) | 61.1 (10.8) | 0.911 |  | 61.4 (11.0) | 59.7 (11.8) | 0.183 |
| Troponin I, mean (SD) mg/dL | 9.5 (15.0) | 11.5 (14.7) | 0.092 |  | 10.2 (15.7) | 10.9 (17.4) | 0.724 |
| Creatine kinase -MB, mean (SD) mg/dL | 16.8 (38.2) | 19.7 (33.3) | 0.333 |  | 18.1 (39.1) | 16.6 (35.5) | 0.736 |
| p-values were determined using t-tests or χ2 tests as appropriate.  Bold style indicates statistical significance (p-value<0.05). | | | | | | | |
